# Fetal ischemia monitoring with in vivo implanted electrochemical multiparametric microsensors

**DOI:** 10.1186/s13036-021-00280-7

**Published:** 2021-12-20

**Authors:** Samuel Dulay, Lourdes Rivas, Laura Pla, Sergio Berdún, Elisenda Eixarch, Eduard Gratacós, Miriam Illa, Mònica Mir, Josep Samitier

**Affiliations:** 1 Nanobioengineering group, Institute for Bioengineering of Catalonia (IBEC) Barcelona Institute of Science and Technology (BIST), 12 Baldiri Reixac 15-21, 08028 Barcelona, Spain; 2 Fetal Medicine Research Center, BCNatal. Hospital Clínic and Hospital Sant Joan de Déu, Universitat de Barcelona, Building Helios 2, Sabino Arana Street 1, 08028 Barcelona, Spain; 3 Institut d’Investigacions Biomèdiques August Pi i Sunyer (IDIBAPS), Barcelona, Spain; 4 Biomedical Research Networking Center in Bioengineering, Biomaterials, and Nanomedicine (CIBER-BBN), Monforte de Lemos 3-5, Pabellón 11, 28029 Madrid, Spain; 5 Department of Electronics and Biomedical Engineering, University of Barcelona, Martí i Franquès 1, 08028 Barcelona, Spain

**Keywords:** Implantable sensor, Ischemia detection, Electrochemical biosensor, pH and oxygen detection, In vivo validation, Tissue and vascular monitoring

## Abstract

Under intrauterine growth restriction (IUGR), abnormal attainment of the nutrients and oxygen by the fetus restricts the normal evolution of the prenatal causing in many cases high morbidity being one of the top-ten causes of neonatal death. The current gold standards in hospitals to detect this relevant problem is the clinical observation by echography, cardiotocography and Doppler. These qualitative techniques are not conclusive and requires risky invasive fetal scalp blood testing and/or amniocentesis. We developed micro-implantable multiparametric electrochemical sensors for measuring ischemia in real time in fetal tissue and vascular. This implantable technology is designed to continuous monitoring for an early detection of ischemia to avoid potential fetal injury. Two miniaturized electrochemical sensors were developed based on oxygen and pH detection. The sensors were optimized in vitro under controlled concentration, to assess the selectivity and sensitivity required. The sensors were then validated in vivo in the ewe fetus model, by means of their insertion in the muscle leg and inside the iliac artery of the fetus. Ischemia was achieved by gradually obstructing the umbilical cord to regulate the amount of blood reaching the fetus. An important challenge in fetal monitoring is the detection of low levels of oxygen and pH changes under ischemic conditions, requiring high sensitivity sensors. Significant differences were observed in both; pH and pO_2_ sensors under changes from normoxia to hypoxia states in the fetus tissue and vascular with both sensors. Herein, we demonstrate the feasibility of the developed sensors for future fetal monitoring in medical applications.

**Supplementary Information:**

The online version contains supplementary material available at 10.1186/s13036-021-00280-7.

## Introduction

Intrauterine growth restriction (IUGR) is a gestational condition in which the fetus does not grow properly during pregnancy. IUGR is the most common cause of human fetal morbidity and mortality [6], affecting 0.7-1.2 million newborn per year, which represents 23% of infant mortality worldwide [18]. Several causes of IUGR have been described, including maternal, genetic, or infectious causes [19, 21, 26]. However, the most important contributing factor to IUGR is a deficient placenta with a low capacity to transport nutrients and oxygen to the fetus, which leads to a decrease in the rate of fetal growth [21]. In the clinical setting, IUGR is suspected when fetal biometrics obtained by routine fetal ultrasound estimates a fetal weight below the 10th centile [24]. Depending on the severity of placental insufficiency, the severity of fetal hypoxia-acidosis will differ. Severe placental insufficiency may be related with a significant hypoxia that if persist during time may induce fetal anabolic metabolism followed by fetal hypoxemia and acidosis [23]. Fetus tries to compensate this situation by redistributing the blood flow to vital organs such as cerebrum and myocardium [29]. Doppler ultrasound would identify these hemodynamical changes [2] which in turn are correlated with the level of oxygen content in blood [4]. When a significant Doppler abnormality is detected, finalization of the pregnancies is indicated to avoid fetal demise [10]. However, Doppler does not allow early detection and continuous monitoring of the metabolic status of the fetus. The description of a device capable of monitoring continuously high-risk pregnancies would allow us to early identify significant hypoxia and acidosis situation. In this direction, a miniaturized implantable sensor for ischemia monitoring in fetal tissue can promptly alert about this problem.

There are different types of tissue ischemia sensors reported that may help in this purpose, utilizing electrochemical [33] and optical techniques such as fluorophore probes [7, 16] and magnetic resonance imaging (MRI) [12, 32]. The MRI offers quantitative information on the pH level, but it is quite expensive, cumbersome due to its lack of portability, and rarely used for real-time monitoring. Fluorophore probes allow high sensitivity tissues bioimaging, but this technique requires the injection of fluorescent molecules, which is not recommended in the fetus and does not allow long-term real time monitoring. Electrochemical sensors is the technology that offers better advantages for implantable applications [13]. This type of sensors permits microfabrication of multiparametric sensors at low cost, maintaining good selectivity, sensitivity, fast response, and reusability for a continuous monitoring in the tissue [17]. Different strategies of analytes detection have been reported for ischemia monitoring in tissue with electrochemical techniques. The most direct is the detection of oxygen deficiency, achieved by the amperometry of oxygen redox at an applied voltage [25]. Other approaches were based on detection of the consequence of the lack of oxygen in the cell. Under hypoxia, the cell undergoes anaerobic respiration with production of lactic acid resulting in a reduction in pH. Electrochemical pH sensors for tissue detection are based on solid-state ion-selective electrodes (ISE), for measuring proton concentration by means of selective ionophores [15]. The reduced oxygen also generates a decrease on the cell energy (Adenosine Triphosphate) with the resulting of cell ions pumping dysfunction, increasing the concentration of sodium and potassium ions in the tissue that are detected by selective ISE sensors [28]. This reduction of ions within the cell causes the entry of water into the cell by osmosis, resulting in a change in cell resistance, which can be monitored by bioimpedance [27].

However, monitoring for ischemia in fetal tissue has inherent difficulties due to the complex in vivo protocol in fetal analysis that can lead to fetal instability and the lower oxygen content compared to adult tissue. The arterial blood in adults’ transports 100 mmHg of oxygen, reaching the muscle tissue around 30 mmHg. Meanwhile the amount of oxygen that crosses the umbilical cord is reduced to 30 mmHg of oxygen and absorbed by the fetus tissue around 15-20 mmHg, being critical hypoxic values of below 12 mmHg [5]. A sensor with high detection sensitivity in a short working range is necessary to be able to discern the low changes of oxygen concentration between normoxia and hypoxia in fetal tissue. This technological challenge makes it difficult to find devices dedicated to ischemia fetal monitoring, being all the aforementioned examples only tested on adult animals and no examples of implantable electrochemical sensors tested in fetus having been reported in the literature.

**Fig. 1:**
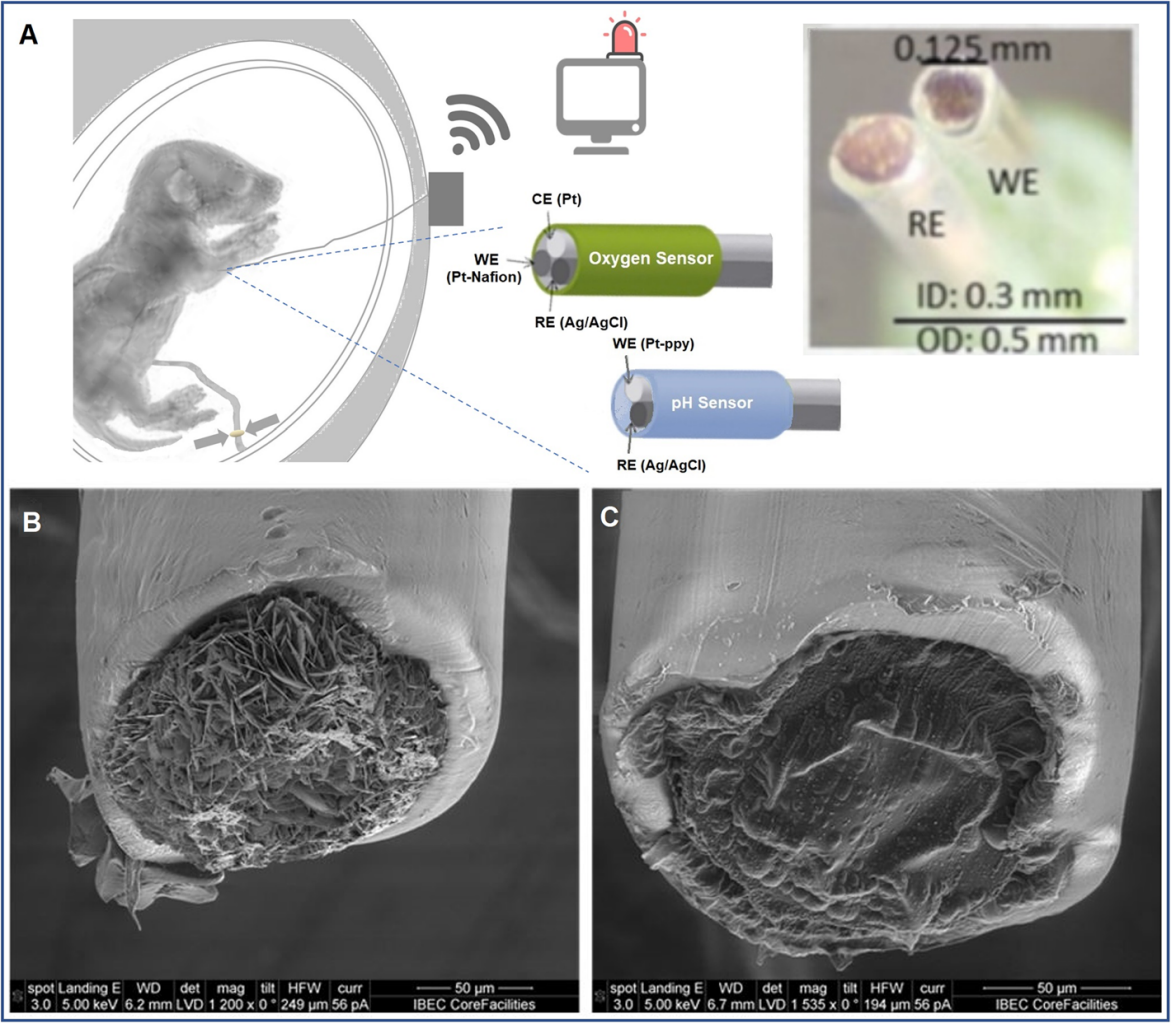
**A** In vivo and developed technology described in this work. On the right, schematic of the in vivo with the umbilical cord occluder and the implanted sensors in the fetus leg. On the right scheme of the oxygen and pH sensors and picture of the pH sensor. **B** SEM image of the modified reference Ag/AgCl electrode and **C** SEM image of the modified working electrode for the pH sensor with a Pt-PPy film

In this work, we describe the in vivo study of micro-implantable multiparametric electrochemical sensors for measuring real time ischemia in fetal tissue and in vascular blood. For this purpose, a 500 μm diameter array was developed, integrating two types of miniaturized electrochemical sensors: an amperometric oxygen sensor and a potentiometric ISE sensor for pH monitoring. The intramuscular implantable sensor permits an early detection of the problem and the multiparametric detection offers more information to the medical doctors regarding the different stages of tissue ischemia; the initial lack of oxygen followed by the tissue’s response to that anaerobic condition resulting in tissue acidosis. The sensors were developed and optimized in vitro under controlled concentration of pH and oxygen, to assess the required selectivity and sensitivity for both sensors and then validated in vivo. The fetal ewe model was selected for this purpose as pregnant ewes share metabolic functions, nutrient transport, and placental physiological characteristics with humans, and due to fetal size, that allow surgical instrumentation [1, 14]. For in vivo validation, the sensors were implanted in the fetal leg muscle and inside the iliac artery. Fetal ischemia was generated by a gradual obstruction of the umbilical cord following an umbilical cord occlusion (UCO) reducing the flow of nutrients and oxygen to the fetus aiming to reproduce perinatal asphyxia described in human pregnancies (Fig. 1A).

## Materials and methods

### Materials, reagents and instrumentation

Potassium chloride (KCl), pyrrole 98%, Nafion® perfluorinated resin 5 wt.%, phosphate buffered saline (PBS) and 500 μm diameter polyetheretherketone (PEEK) microtubing’s for electrodes assembling were purchased from Sigma-Aldrich (Spain). The electrodes were constructed with metal 125 μm diameter wires (platinum (Pt), silver (Ag), copper) at 99.9% purity, insulated with polytetrafluoroethylene and purchased from Advent Research Materials (UK). Ultrapure water from Milli-Q systems (Millipore).

Electrochemical measurements were performed with a portable custom-built multipotentiostat from PalmSens (The Netherlands). The pH sensing was tested in a standard commercial pH solution of 4.006 and 7.413 (Mettler-Toledo S.A.E, Spain) for pre-calibration and sensor characterization. Pre-calibration for dissolved oxygen concentrations in PBS buffer was measured using a PreSens oxygen meter (Germany).

Scanning electron microscope (SEM) characterization was performed with a NOVA NanoSEM 230 from Thermofisher Scientifics (USA).

### Sensors preparation

#### pH sensor preparation

The pH sensor consisted of two electrode systems: a polypyrrole (PPy) modified Pt working electrode (WE) and an as pseudo reference electrode (PRE). 0.1 M distilled pyrrole and 0.1 M KCl in aqueous solution was used for film formation by electro polymerization process by cycling the Pt electrode between 0.45-0.95 V three times at 0.025 mV s-1. The Pt/PPy electrode was preconditioned in 0.1 M HCl solution for 48 h prior to use. PREs was constructed by immersing the tip of the Ag wire (anode) and a corresponding Pt or Ag wire (cathode) in 3 M KCl for 2 min using 9 V battery, which resulted in the formation of Ag/AgCl layer. The PREs were then coated with Nafion® and cured at 100 °C for 1 h. Both electrodes were inserted within a piece of 5 mm PEEK tubing and stored under argon [8]..

#### Oxygen sensor preparation

The oxygen sensor consisted in three electrodes: bare Pt wire as counter, Ag/AgCl PRE and membrane-modified Pt wire as a WE. The Pt wire was prepared as WE with two drops of Nafion® as received on the metallic surface and dried overnight in an argon and then cured at 100 °C for 1 h. The PRE was prepared as described above. The prepared electrodes were inserted in the PEEK tubing as described above. Just before using, the modified sensors were swelled in PBS for 20 min [25]..

### Electrochemical pH and O_2_ monitoring

500 μm diameter PEEK microtubings was used for electrodes assembling.

All in vitro pH calibrations were performed with standard commercial pH solutions of 4.006 and 7.413. Calibrations for O_2_ sensing, were performed with PBS buffer with different oxygen concentration by purging with N2 or O_2_ for at least 30 min.

For continuous pH sensing, potential difference between electrodes was recorded every 0.1 s. For O_2_ monitoring, chronoamperometry (CA) was performed at fixed − 0.7 V potential for 300 s. PS Trace 4.8 was used as electrochemistry software and data collection.

### In vivo studies in ewe as animal model

#### Animal instrumentation

A total of 8 Ripollesa pregnant ewes with gestational ages between 115 and 125 days (term 147-150 days) were included in this study. Animals were provided by a certified commercial farm and all experimental procedures were performed in accordance with applicable regulation and guidelines and with the approval of the Animal Experimental Ethics Committee of the University of Barcelona (ref 214.17 and the competent authority Generalitat de Catalunya (ref 9645)).

Animals were premedicated with ketamine, xylazine and midazolam (4 mg/kg, 0.2 mg/kg and 0.2 mg/kg respectively, IM) and 2-4 mg/kg (IV) of propofol was administered for induction anesthesia. Animals were intubated for mechanical ventilation. Buprenorphine (0.02 mg/kg, IV) and progesterone (150 mg, SC) were administered before surgery. Infraumbilical midline laparotomy was performed for uterus exteriorization and hysterotomy was executed for fetal exteriorization.

Fetal hindlimbs were exteriorized and fentanyl (0.2 ml, IM) was administered for fetal analgesia. A vascular occluder (OC20HD, UNO Roestvaststaal BV) connected to a 10 ml syringe loaded with saline was placed around the umbilical cord. Afterwards, catheterization of the left iliac artery for serial blood sampling and the insertion of a pH sensor for blood monitoring in the left iliac artery was performed. Thereafter, 1-2 oxygen sensors and 1-2 pH sensors were inserted in the right and left femoral quadriceps respectively. Fetal hind limbs were interiorized inside the uterus and hysterography was then performed using a running suture. Sensor wires, iliac vascular catheter and the occluder tubing were exteriorized through the uterine incision.

#### Umbilical cord occlusion (UCO)

To induce fetal tissue hypoxia and acidosis in a progressive and controlled manner, an umbilical cord occlusion (UCO) protocol was followed. The UCO protocol was divided in four different stages: basal, 50% occlusion, 100% occlusion and recovery (without occlusion). For the basal and recovery stages the occluder was deflated and the blood flow from the umbilical cord was passing in a physiological manner. For the occlusion, the occluder was inflated to obliterate the umbilical cord and reduced the blood flow. For the 50% stage, the occluder was filled with 1.5 ml of saline and with 3 ml to induce a complete occlusion.

At different intervals, 0.2 mL of blood samples from the fetal iliac artery catheter were extracted and analyzed for gasometry with standard equipment (EPOC® reader and EPOC BEGM test card, Alere/Siemens healthcare, Germany). The blood sample and sensor signal were recorded at the basal stage every 10 min for 20 min. Average of the measures were reported in this work as Pre-occlusion. Then, the umbilical cord passage was occluded at 50% and recordings were obtained every 10 min for 20 min (50% occlusion) and at 100% occlusion recordings were obtained every 5 min for 10 min (100% occlusion). Finally, the occluder was opened for recovery of the initial umbilical cord flow and recordings were obtained every 10 min for 20 min. The average of the measures was reported as Recovery.

After the occlusion protocol, animals were euthanized with pentobarbital (200 mg/kg, IV). Death was confirmed by the cessation of circulation and breathing in both ewes and fetuses.

### Statistical analysis

Graphics and statistics analyses were performed in GraphPad 9.1. Potentiometric and chronoamperometric data was normalized to reduce the variability between sensors. Oxygen partial pressures were reported primarily in mm Hg to compare with EPOC® device’s units, used as our standard. Oxygen unit conversion was carried out with a tool provided by PreSens (PreSens, n.d.).

Interquartile range rule (IQR) was used for identifying outliers. One-way analysis of variance (ANOVA) was used to evaluate the statistical differences between multiple results. With a confidence value of α = 0.05, values *p* > 0.05 were considered statistically not significant (ns); those between 0.01 < *p* < 0.05 were considered significant (*); 0.001 < *p* < 0.01 were very significant (**); 0.0001 < *p* < 0.001 were extremely significant (***) as well as those *p* < 0.0001 (****). t-test was used to assess statistical differences between two sample means (two-tailed paired or unpaired, when appropriate). In vitro data were expressed as mean and standard deviation error (SD) and in vivo data as mean and standard error of the mean (SEM).

## Results and discussion

### In vitro optimization

In this study, we aimed to monitor ischemia by measuring pH and oxygen content in tissue and vascular to the ewe fetus after hypoxia-ischemia induction in sheep fetus following a progressive UCO. Prior to in vivo studies, in vitro optimization was required to optimize and characterize both sensors under controlled conditions. Firstly, the sensors were fabricated and specifically functionalized for oxygen or pH sensing.

#### pH sensor in vitro optimization

The working principle of the pH sensor is due to the specific adsorption of protons in the PPy and the deposited cations are measured by differential potential with respect to a PRE in the vicinity of the pH sensor. Figure 1B and C shows respectively SEM images of the successfully modified tips of PRE and the pH WE. In Fig. 1B can be appreciated the grown silver chloride crystals on the electrode, showing the rough surface with nanostructured-type crystals silver chloride on the silver surface covered with a layer of Nafion®. Whilst Fig. 1C depicts a black homogenous PPy film formed on Pt WE by electro polymerization.

**Fig. 2:**
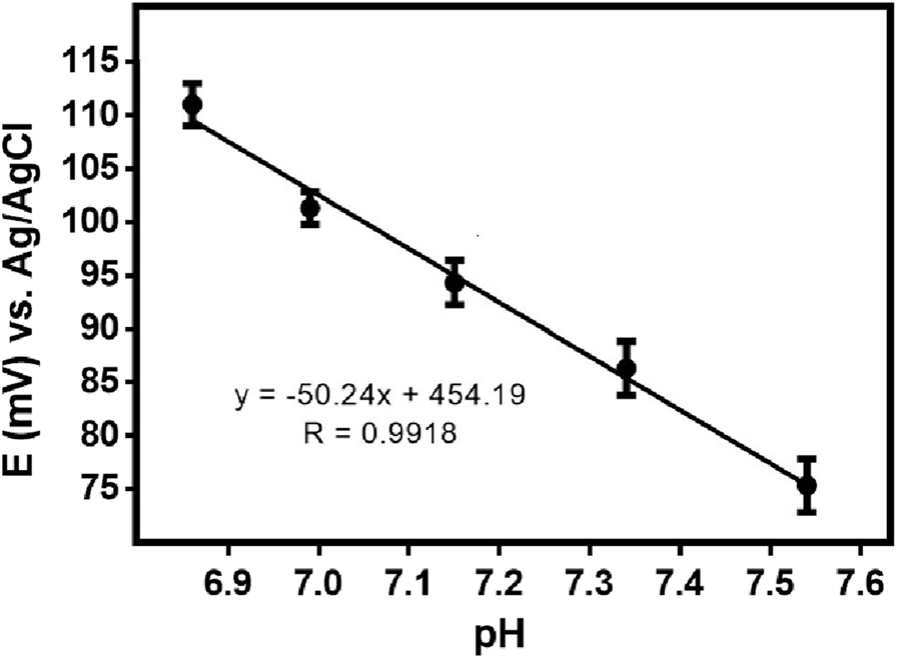
pH sensor electrochemical characterization of calibration plot obtained from buffer solution under physiological pH values. Data are expressed as Mean ± SD

The potentiometric ISE sensors characteristic is related with the slope in the linear regression under different concentrations. The developed pH ion-selective solid-based micro-sensor exhibited a stable near Nernstian response of − 50 mV/pH unit when used in physiological range of pH 6.8-7.5. The pH calibration plot also demonstrated a wide working linear range, maintaining sensitivity in a broad pH range from 4 to 9 [8] (Fig. 2).

Selectivity of the pH sensor was evaluated by mixed solution method [9], considering cations similar than the target of interest found at higher concentrations in physiological samples; Na + and K+ [3, 30]. The developed pH sensor exhibited low interference activity of the tested interference ions, obtaining selectivity coefficient (KAB) values of 5.37 × 10-2 for Na + and 1.12 × 10-2 for K+ [8]. This further suggests that there is not a great impact of the ionic strength of other endogenous cations and the developed pH sensor remained selective for H+.

Since the pH of any solution is a function of its temperature, all tests and calibrations performed are carried out at 37 °C to imitate physiological conditions. The response time (t90%) of the sensor towards a step change in pH is 20 s per decade in all cases. Moreover, the developed pH sensor exhibited a reversible response upon switching from low to high or high to low pH samples (Fig. 1S, see supporting information).

#### Oxygen sensor in vitro optimization

Oxygen sensor was optimized as previously reported [25]. Briefly, in vitro optimizations were performed electrochemically in PBS and stored blood with different oxygen content. The working range of the O_2_ sensors in PBS were between 5.5 (0.75%) to 742 (100%) mmHg at 25 °C, giving a sensitivity of − 2.50 nA/mmHg.

In our previous study, we showed that O_2_ Pt-Nafion sensor (vs. Ag/AgCl) exhibits a E1/2 at approximately − 0.6 V, and at this potential other interferents such metal ions, cysteine or certain anesthesia gases as nitric oxide and isoflurane, may be active close to this potential. However, the efficient combination of the hydrophobic fluorinated backbone with the hydrophilic sulfonic acid groups within the Nafion® membrane, contributes to restrict the transport of certain interfering species. The absence of extra redox peaks when blood at low oxygen content was tested by cyclic voltammetry, showed that if there were changes in the signal due to interfering molecules, these were negligible and had no effect on the selectivity of the sensor towards O_2_ in tissue [25]..

### Hypoxia fetal monitoring in vivo

The sheep species was selected for this in vivo study mainly for two reasons. The first is due to the large size of the sheep fetus (similar to the human fetus), which allows efficient insertion of electrochemical sensors into fetal tissue and the placement of catheters to facilitate serial blood sampling. The second is the similar fetal blood gas measurements between humans and sheep [22]. Furthermore, the above OCUs in pregnant ewes were standardized and easy to reproduce for our purposes [11, 20, 31]. Once the model’s safety has been optimized and ensured, in a second step, the performance of these electrochemical sensors in complicated pregnancies in humans must be evaluated.

**Fig. 3:**
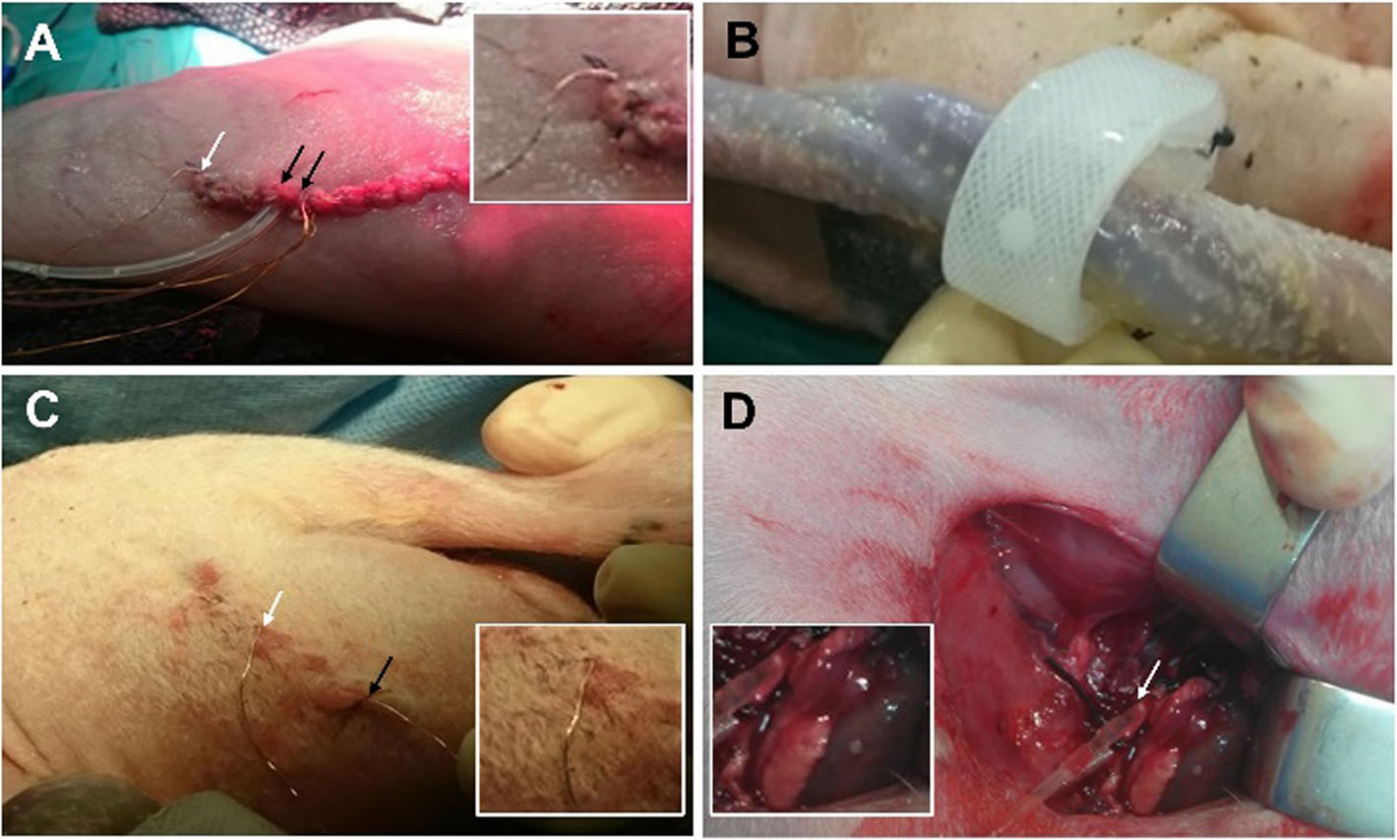
**A** Ewe uterus containing the fetus with the sensors implanted in the fetus leg pointed out by arrows. **B** Occluder placed around the umbilical cord of the ewe to control the passage of oxygen to the placenta **C** Muscular implanted sensor in the ewe fetus leg pointed out by arrows. **D** Vascular implanted pH sensor in the iliac artery of the ewe fetus (white arrow). The inlets show the zoom area indicated by the white arrow

The micrometric sensor was designed for fetal application and shaped to be inserted by means of a needle that can be later withdrawn, leaving the sensor implanted. To evaluate the functionality of the sensor and the feasibility of ischemia detection in fetus, the sensor insertion in muscular tissue and inside vascular vessels was at that stage performed through a surgical incision, instead of needle insertion. The sensors were inserted in the ewe fetus at the femoral fetal quadriceps muscle tissue on the leg (Fig. 3A) and inside the iliac artery (Fig. 3B) for continuous monitoring of tissue and blood ischemia in vivo, respectively. Then, hysterography was performed allowing the exteriorization of external cables of the sensors (Fig. 3C) and the sensors were externally connected with a portable wireless device that allows the continuous monitoring of both sensors in real time. Sensors monitoring started with normoxia conditions and hypoxia was induced by the gradual UCO (Fig. 3D).

Since there is not yet a commercially available sensor device for standard intramuscular tissue measurements, a standard equipment (EPOC®) for blood measurements ex vivo was considered as a reference for performance evaluation of the developed micro-sensors in vascular and intramuscular monitoring, under conditions of pre-occlusion (normoxia) and occlusion (hypoxia) during in vivo measurements.

#### Intravascular pH monitoring

**Fig. 4:**
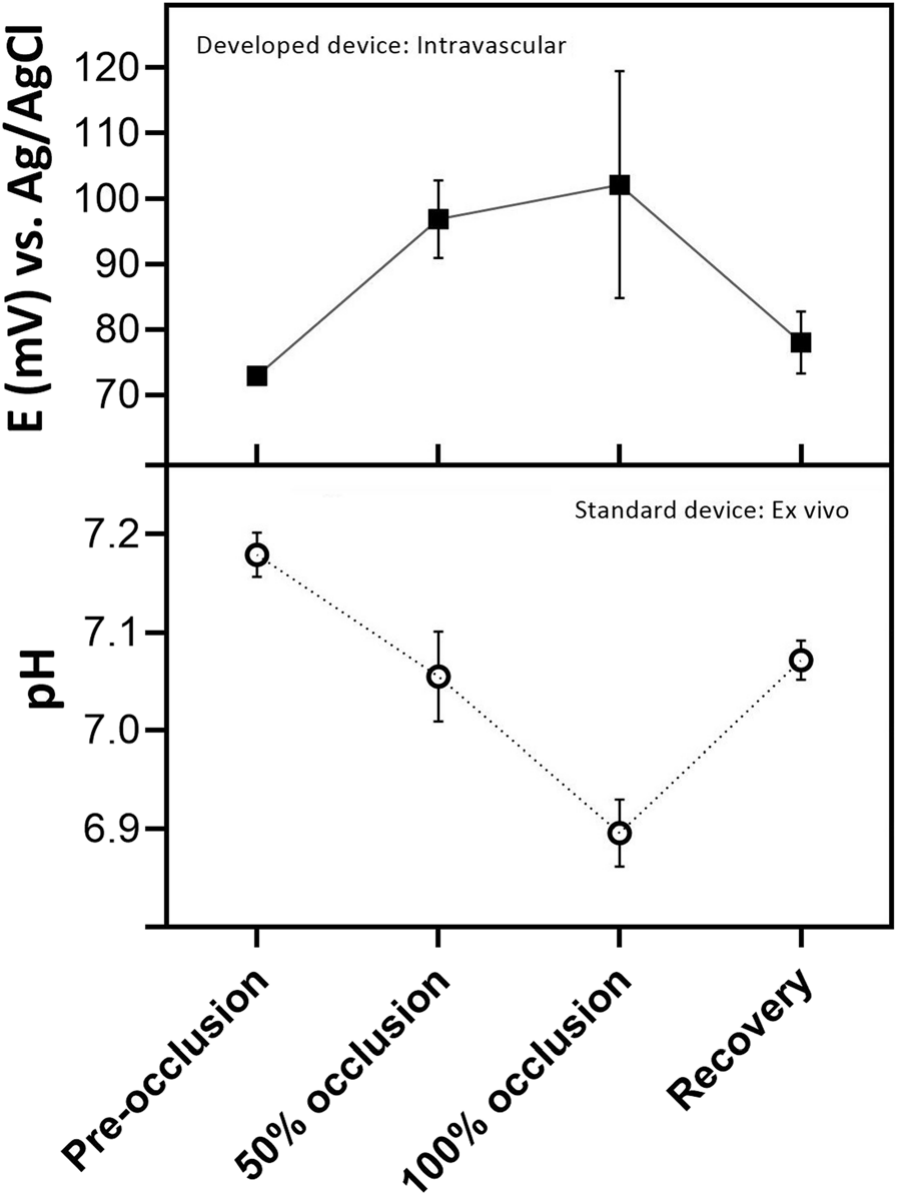
Correlation of obtained pH results in ischemia analysis of the ewe fetus under different degrees of umbilical cord occlusion, using the developed device in vivo by vascular insertion (*n* = 3 sensors, *N* = 3 ewe fetuses) and the standard device by ex vivo blood extraction (n = 8 sensors, N = 8 ewe fetuses). Data are expressed as Mean ± SEM

Intravascular pH monitoring was performed simultaneously with intramuscular monitoring by inserting a sensor into the left iliac artery of the ewe fetus. In this case, few repetitions (*n* = 3 sensors, *N* = 3 ewe fetuses) could be performed due to the complexity of the surgical insertion in the vascular system to limit the number of jeopardized experiments. The fetus has a reduced diameter of the artery and rapid instability of the fetus with little blood loss. Thus, vascular measurements were limited to triplicate, so there is not enough data to perform a one-way ANOVA statistical analysis. Figure 4 shows the results obtained intravascularly in vivo in ewe fetuses under different respiration conditions with the developed pH sensor and the response obtained with the blood test (ex vivo) (*n* = 8 sensors, *N* = 8 ewe fetuses) with the standard device under the same conditions.

The intravascular results show a clear difference from the umbilical cord pre-occlusion stage to the partial occlusion, and reduced differences from 50% to total occlusion. When the blood flow is recovered, the pH sensor inserted in the vascular system can detect the recovery to the basal pH, demonstrating a good performance of the developed pH sensor in the fetal vascular system. Comparing the results obtained in blood extraction with the standard equipment and with the developed sensor, intravascularly implanted in vivo, it has been observed that there is good degree of correlation with the standard device (Fig. 4).

Under conditions of total occlusion of the umbilical cord, comparable values are reached with both equipment. However, if the analysis response is observed in which there is less change in pH conditions due to ischemia; as it is after applying 50% occlusion and once the normoxic conditions have been recovered, a greater response of the developed device is observed. This difference can be due to different factors; One factor may be a greater sensitivity of the sensor developed in the detection of low pH variation, therefore the change observed at low concentrations with the standard equipment is less. Nevertheless, the differences affect a pH range of 0.2 and the EPOC® device can achieve this sensitivity. So, it may be other variables.

Since blood is a living sample and continuously uses O_2_ to produce CO_2_ and lactic acid with a change on pH, it must be analyzed immediately for accurately reflect the status of the blood analytes. The ex vivo analysis protocol took approximately 1 min from the extraction of the blood sample to its insertion into the EPOC®. Device instructions recommend testing samples immediately, if possible, but they can be stored at room temperature for up to 30 min. Taking this information into account, the time factor may be negligible considering the short time of extraction used.

Another factor that may affect is the presence of dead volume in the blood collection catheter, which means that low volume of the blood sample from the previous stage is present in the next blood sample collection and can dilute the next collected blood samples, minimally distorting the true value in the sample.

All the factors discussed influence the precision of the blood analysis, being more efficient and dynamically faster the in-situ analysis in the vascular system performed with the developed device.

In addition, considering the final application of the sensors developed for the continuous monitoring of high-risk pregnancies with the possibility of suffering IUGR, these sensors allow the identification of significant hypoxia and acidosis situations as standard methods do, but with an implanted sensor, which makes early detection of the problem more feasible.

#### Fetus intramuscular in vivo pH monitoring

**Fig. 5:**
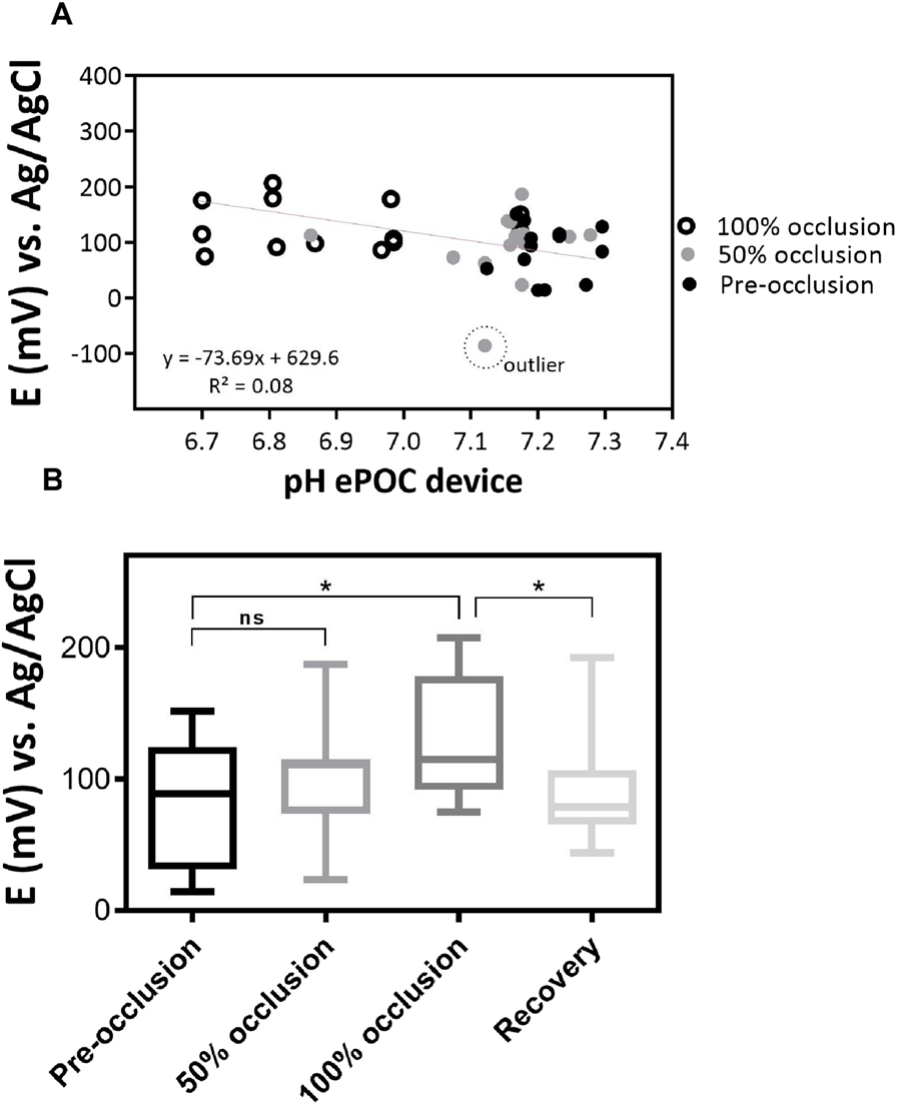
Correlation of potential difference obtained from the pH sensor with the standard device in the real-time monitoring of intramuscular ewe leg tissue under different umbilical cord occlusion (pre-occlusion, 50% occlusion, 100% occlusion, and recovery), **A** Scatter plot, and **B** Box-plot presenting the analysis of variance (one-way ANOVA) between different stage of the ewe fetus umbilical cord occlusion for the intramuscular pH monitoring with the developed device (*n* = 7 sensors, *N* = 8 ewe fetuses). Statistical significance was declared when *p < 0.05, between pre-occlusion and each occlusion state

The performance of the pH micro-sensor was evaluated in muscular tissue of ewe fetus. The difference of potential observed from the pH sensors inserted intramuscularly in the ewe fetus leg under the different occlusion stages was correlated with the pH values obtained with the EPOC® standard device with the extracted blood under the same occlusion stages. The calibration plot in Fig. 5 shows the correlation and the extrapolation of the value obtained by the developed pH sensor and in the standard device. The increase of voltage signal with the pH sensor, indicates the presence of higher concentration of protons on the sensor and so, an acidic pH on the tissue, which occurs under ischemic conditions. The changes of voltage due to the occlusion evolution of the ewe umbilical cord in Fig. 5, and the excellent correlation with the standard device shows the good performance of the developed sensor. From the standard measurements, a pH range was determined for the ewe fetus stage before occlusion between 7.30 to 7.17, under 50% of occlusion between 7.17 to 7.05 and for the complete occlusion below 7.05, reaching pH values of 6.70.

Figure 5A shows the potential differences obtained with the intramuscularly implanted pH sensor in correlated with the pH values obtained from the standard blood-based device, giving a sensitivity of (− 79.77 ± 0,51) mV/pH.

The statistical results in the one-way ANOVA (α = 0.05) of the obtained data during animal experimentation for intramuscular pH measurements, show a significant difference in the pre-occlusion values (normoxia) with respect to those of 100% occlusion, with a significance level of *p* = 0.0105, not observing significant differences at 50% occlusion. Similar significant differences are observed from the pH sensor when the umbilical cord occluder was opened and left to flow again oxygen (recovery period) with a *p*-value of *p* = 0.020, demonstrating a good performance and sensitivity of the pH sensor under the complexity of this fetal tissue in vivo experiment and the low differences between normoxia and hypoxia pH in fetus (Fig. 5B).

#### Fetus intramuscular in vivo O_2_ monitoring

Simultaneously to the intramuscular monitoring of pH sensors, oxygen sensors follow the same in vivo process and correlation with the standard blood-based device were performed. The same device could be used, since EPOC® is able to analyze pH, Na^+^, K^+^, Ca^+ 2^, Cl^−^, pO_2_ and pCO_2_ in blood. The oxygen ranges under the different conditions of occlusion of the umbilical cord in ewe fetuses were determined with the standard device, observing oxygen concentrations higher than 18 mmHg for the pre-occlusion stage, between 18 and 12 mmHg under 50% occlusion and below 12 mmHg completely occluding the cord.

**Fig. 6:**
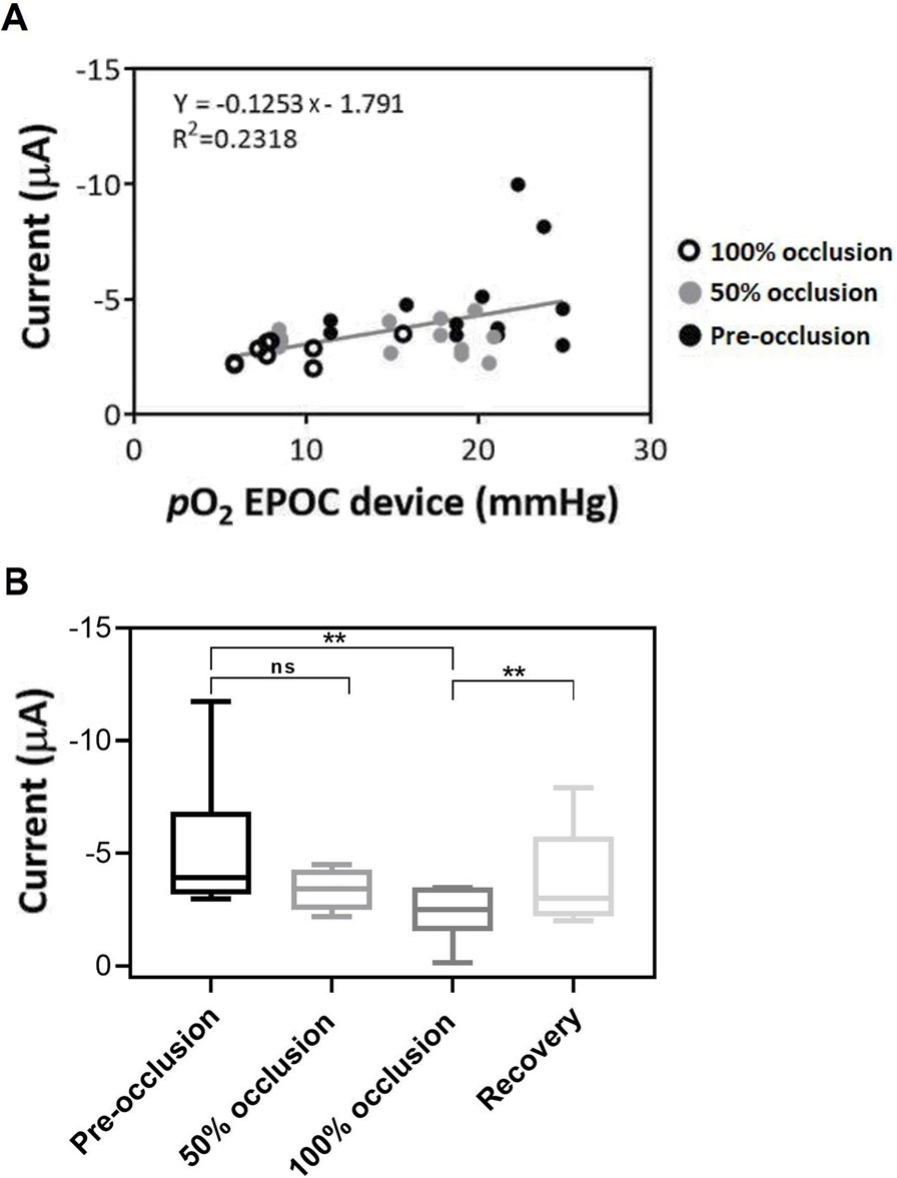
pO_2_ concentration in in vivo studies of ewe fetus intramuscular tissue under different respiration stages. **A** Correlation between cathodic current obtained with the developed sensor and pO_2_ mmHg concentration from standard device in the range of 0-30 mmHg. **B** Boxplot showing the analysis of variance (one-way ANOVA) between different stage of the ewe fetus umbilical cord occlusion for the intramuscular oxygen monitoring with the developed device (*n* = 11 sensors, *N* = 8 ewe fetuses). Statistical significance was declared when *p < 0.05, **p < 0.01 between pre-occlusion and each occlusion state

The cathodic currents of each stage of occlusion in the in vivo analysis of intramuscular tissue of ewe fetuses were plotted versus the blood ex vivo pO_2_ obtained with the EPOC® device, giving a sensitivity of (− 0,12 ± 0,04) μA/mmHg (Fig. 6A).

The results obtained with the developed oxygen sensor were statistically analyzed with a one-way ANOVA. The different stages of umbilical cord occlusion of ewe fetus and the subsequent variations of oxygen concentration in the muscular tissue were clearly detected. The results showed high significant differences between pre-occlusion and 100% occlusion obtaining a *p*-value of *p* = 0.0041, not observing significant differences at 50% occlusion. However, the statistical *p* value obtained at 50% (*p* = 0.0520) is very close to the cut-off value of α = 0.05. After the recovery of the initial blood flow through the umbilical cord, the oxygen signal is restored with significant differences with respect to 100% occlusion with a p-value of 0.0018. On the other hand, no significant difference was found between occlusion at 50 and 100% (*p* = 0.0864) as depicted in Fig. 6B. These statistical results showed that our developed O_2_ sensor was successfully monitoring changes of pO_2_ concentration in the fetus tissue while the occlusion protocol was ongoing, meaning that it would be potentially useful in monitoring the O_2_ tissue of fetus.

#### Fetus intramuscular multiparametric analysis

**Fig. 7:**
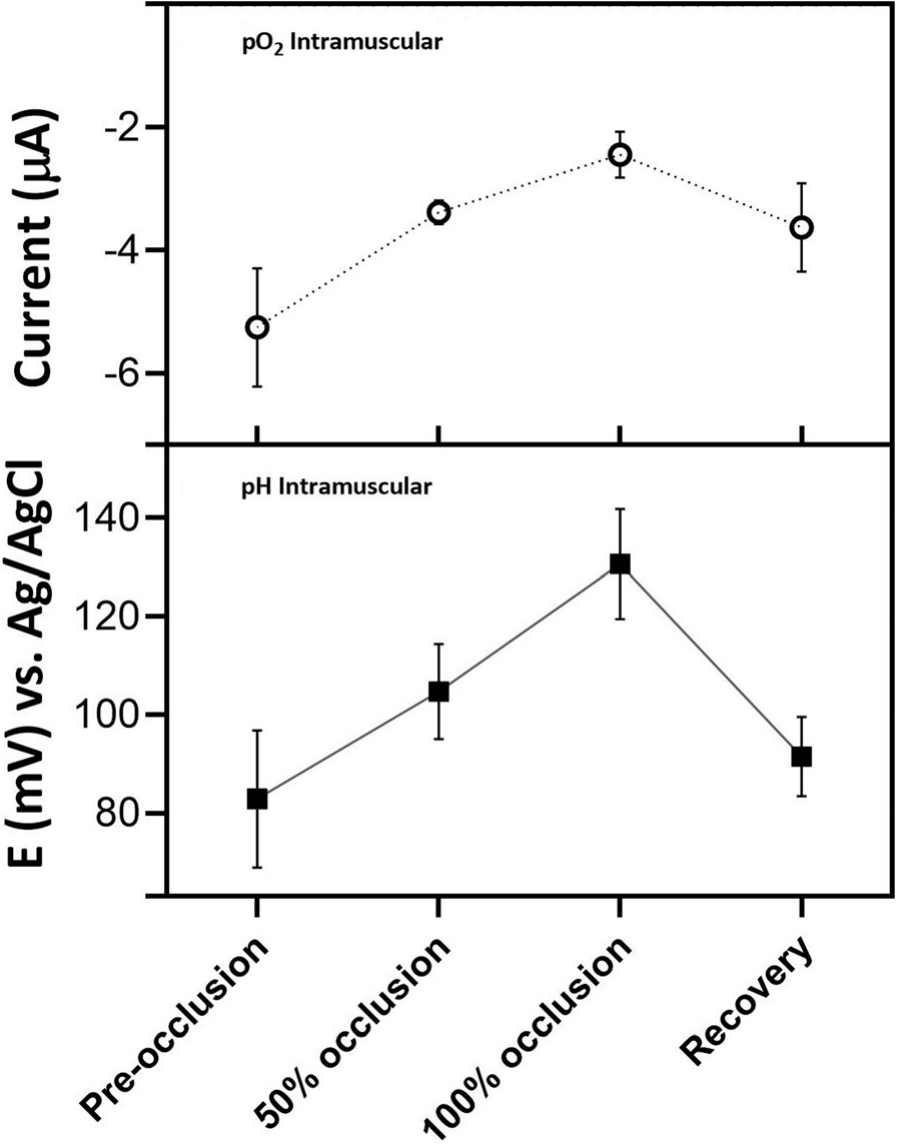
Multiparametric analysis of ischemia in vivo of the ewe fetus under different degrees of umbilical cord occlusion with the oxygen sensor (*n* = 11 sensors, *N* = 8 ewe fetuses) and the developed pH sensors (*n* = 7 sensors, N = 8 ewe fetuses). Data are expressed as Mean ± SEM

The electrochemical miniaturized device allows the integration of both sensors in a final diameter of 500 μm. The implantation of pH and oxygen sensors in the ewe fetal tissue permit the simultaneous analysis of both analytes in real time (Fig. 7). Comparing the results obtained with the two implanted sensors, both have a similar trend response. Thus, when the O_2_ concentration decreases, detected by the lower reduction current of the oxygen sensor, the pH sensor shows an increase in voltage, due to a decrease in pH on the sensor surface. The multiparametric sensor provides information on the oxygen content in the tissue and the tissue acidosis, because of the lack of oxygen.

The progressive reduction of oxygen transport through the umbilical cord is gradually detected with greater differences (higher significant differences between hypoxic and normoxic states) and lower values dispersion (higher R2 in the lineal regression of the measured values) in the oxygen sensor compared to the pH sensor (Figs. 5 and 6). These differences between sensors may be due to a lower sensitivity of the pH sensor, considering the small pH changes in a partial hypoxia in fetus. But also, these differences can be inherent in the analytes to be monitored. In the case of oxygen, the decrease in concentration in the tissues is directly related to the blockage of oxygen flow. However, in the case of pH, this is a consequence of the lack of oxygen, which requires time for the tissues to react for it to occur (anaerobic cellular metabolism secondary to hypoxia) and then it depends on the different body reaction to changes of each measured animal, increasing the dispersion of the response. However, the multiparametric sensor developed with the simultaneous monitoring of these two variables, allows distinguishing different hypoxia stages in the tissues, offering more information to the medical doctor.

No adverse effects were detected in the health of the fetus and eve due to implanted sensors monitoring. The electrochemical sensors are miniaturized and minimally invasive. They were inserted in the hindlimb skeletal muscle which do not affect the fetal wellbeing. Finally, histological assessment of the tissue surrounding the sensors was performed and revealed no signs of tissue alteration on the same day of insertion.

Improvements are needed in the devices to reduce the dispersion of results. In this direction, further research would be focused in designing a fabrication method that could be industrially scalable to reduce variabilities in the manual fabrication. Moreover, a telemetric system for a fully wireless and portable implantable system will be studied. In vivo long-term analysis needs to be performed, considering antifouling methods compatible with the developed sensors. Furthermore, this array of sensors was designed for a simple implantation method with a syringe that needs to be tested in vivo, since open surgery was used for this purpose to reduce variables in this study.

## Conclusions

In summary, a micro-implantable array of pH and pO_2_ sensors has been developed for fetal ischemia/hypoxia monitoring in tissue and vascular. The sensors were tested in vivo in ewe fetal model, generating hypoxia by modulating the umbilical cord occlusion. The developed sensor response for pH and pO_2_ were compared with a standard EPOC® device, using blood samples extracted from the fetus under conditions of normoxia and hypoxia, since is not commercially available a standard intramuscular tissue sensor. In vivo studies with the sensors implanted in the muscular tissue of the fetus demonstrated discrimination between normoxia and hypoxia states, with significant statistical differences with both sensors between pre-occlusion and 100% umbilical cord occlusion with a *p*-value of *p* = 0.0105 and *p* = 0.0041 for pH and pO_2_ sensors respectively. In parallel to the intramuscular measurement in vivo, the pH microsensor was implanted in vivo in the iliac artery of the fetus to analyze the blood hypoxia in situ in the vasculature and it was subjected to the same conditions of fetal respiration of hypoxia and normoxia. The results were compared with the ex vivo analysis with the standard device, observing a similar trend in the response, but a greater sensitivity at low pH values by the equipment developed in this work.

The results obtained with the implanted intramuscular and vascular microsensor are relevant due to the challenge of working with a fetal model. In fetal tissue there is a lower concentration of O_2_ and pH compared to an adult, and therefore, the differences in the parameters analyzed in different respiratory conditions are smaller. In addition to the complexity of the in vivo fetal model, which is much more unstable than the adult animal model.

The developed microsensor technology presented can lead a new generation of implantable diagnostic tools for fetal monitoring to improve fetal surveillance and wellbeing.

## Supplementary Information

**Additional file 1.**

## Data Availability

The datasets analyzed during the current study available from the corresponding author on reasonable request.

## Abbreviations

IUGR: Under intrauterine growth restriction
MRI: Magnetic resonance imaging
ISE: Ion-selective electrodes
PBS: Phosphate buffered saline
SEM: Scanning electron microscope
PEEK: Polyether ether ketone
PPy: Polypyrrole
PSE: Pseudo reference electrode
WE: Working electrode
CA: Chronoamperometry
UCO: Umbilical cord occlusion
IQR: Interquartile range rule
SD: Standard deviation
