# Supplementary material for: Fetal ischemia monitoring with in vivo implanted electrochemical multiparametric microsensors
Source: J Biol Eng. 2021 Dec 20;15:28. doi: 10.1186/s13036-021-00280-7 (PMC8691007; doi:10.1186/s13036-021-00280-7)
Supplement: Supplementary file 1 — Additional file 1. [file 13036_2021_280_MOESM1_ESM.docx]

***Supporting Information***

***Fetal ischemia monitoring with in vivo implanted electrochemical multiparametric microsensors***

Samuel Dulay^1,§^, Lourdes Rivas^1,§^, Laura Pla^4^, Sergio Berdún Marin^4^, Elisenda Eixarch^4.5^, Eduard Gratacós^4.5^, Miriam Illa^4^, Mònica Mir^1,2,3,¥*^ and Josep Samitier^1,2,3,¥^

*^1^ Nanobioengineering group, Institute for Bioengineering of Catalonia (IBEC) Barcelona Institute of Science and Technology (BIST), 12 Baldiri Reixac 15-21, Barcelona 08028, Spain*

*^2^ Centro de Investigación Biomédica en Red en Bioingeniería, Biomateriales y Nanomedicina (CIBER-BBN), Monforte de Lemos 3-5, Pabellón 11, 28029 Madrid, Spain*

*^3^ Department of Electronics and Biomedical engineering, University of Barcelona, Martí i Franquès 1, 08028 Barcelona, Spain*

*^4^ Fetal Medicine Research Center, BCNatal. Hospital Clínic and Hospital Sant Joan de Déu, Universitat de Barcelona. Building Helios 2, Sabino Arana Street 1, 08028 Barcelona, Spain*

*^5^ Institut d’InvestigacionsBiomèdiques August Pi i Sunyer (IDIBAPS), Barcelona, Spain*

§Both authors contributed equally to this work (S.D. and L.R.)

^¥^Equal Senior contribution (M.M and J.S)

*Corresponding author: [mmir@ibecbarcelona.eu](mailto:mmir@ibecbarcelona.eu)


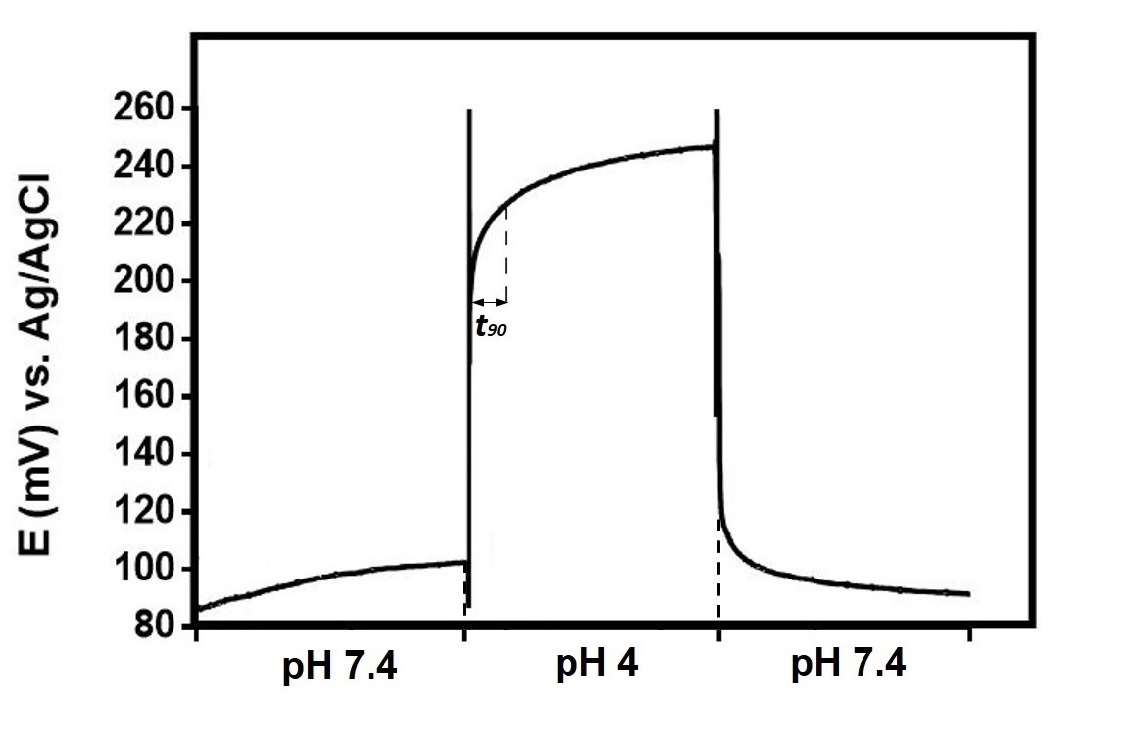


**Figure 1S**. pH sensor electrochemical characterisation of sensor time and reversibility in buffer solution under strong pH variations.
